# A double blind randomized active-controlled clinical trial on the intra-articular use of Md-Knee versus sodium hyaluronate in patients with knee osteoarthritis (“Joint”)

**DOI:** 10.1186/s12891-016-0948-4

**Published:** 2016-02-22

**Authors:** Luis Severino Martin Martin, Umberto Massafra, Emanuele Bizzi, Alberto Migliore

**Affiliations:** Department of Internal Medicine, “Regina Apostolorum” Hospital, Via S. Francesco 50, Albano Laziale, Rome, Italy; Operative Unit of Rheumatology “S.Pietro–Fatebenefratelli” Hospital, Via Cassia 600, 00189, Rome, Italy

**Keywords:** Osteoarthritis, Knee, Intra-articular, Hyaluronic acid, MD-Knee

## Abstract

**Background:**

To evaluate the clinical outcomes of a group of patients affected by knee osteoarthritis (OA) treated with MD-Knee (Guna S.p.a., Milan, Italy) versus a group of patients treated with sodium hyaluronate.

**Method:**

This non-inferiority prospective randomized controlled trial involved 60 patients affected by knee OA, grade 2–3 of Kellgren-Lawrence scale. The MD-Knee Group, Group A (*n* = 29) was administered five intra-articular injections at 1 week interval; the sodium hyaluronate Group, Group B (*n* = 31), was administered five doses of intra-articular injection of sodium hyaluronate at 1 week interval. All patients were prospectively evaluated before and at 3 and 6 months after the treatment by the Lequesne Knee Index (LKI) as primary endpoint and the Visual Analogue Scale (VAS), Pain Killer consumption and SF-36 questionnaires as secondary endpoints.

**Results:**

At the 3- and 6 month follow-up, LKI and VAS improved significantly in both groups compared to baseline and no statistically significant differences were observed between Group A and Group B. There was no statistically significant difference in the SF36 questionnaire score and pain killer consumption between two groups at any time point.

**Conclusions:**

This study shows that both preparations exert similar clinical effects as assessed through multiple outcome measures. MD-Knee is effective on knee OA symptoms over 6 months after a 5-weekly injection course, and it is equally effective as the reference sodium hyaluronate.

**Trial registration:**

Trial registration number: ISRCTN93862496. Registration date: January 18th, 2016

## Background

Osteoarthritis (OA) is a chronic degenerative joint disease characterized by progressive damage of articular cartilage and underlying bone. It is a common rheumatic disease that affects both sexes and the majority of the elderly people; nevertheless, also the young are frequently affected by OA, thus becoming an important cause of lost workdays. Estimated prevalence in general adult population is of 11 and 24 % for hip and knee OA respectively [1]. Pain is accentuated by movements and decreases with rest but, with progression of disease, it may be present at rest and accompanied by short morning stiffness; moreover, joint damage causes a progressive functional limitation [2]. In order to reduce pain and to achieve an overall better clinical condition it is suggested to use a therapeutic strategy including physical therapy and rehabilitation, non-steroidal anti-inflammatory drugs (NSAIDs), analgesics, chondroprotecting agents and intra-articular treatment with infiltrative substances such as hyaluronates and steroids. When the disease is at an advanced stage, orthopedic surgical solution can offer great benefits [3]. Over the past 10 years some double-blind controlled clinical trials have shown that administration by injection of hyaluronic acid (HA) for 3–5 weeks is superior in terms of efficacy, compared to saline, arthrocentesis, and treatments with NSAIDs [4–6]. In addition, HA has presented an excellent tolerability profile with a low incidence of complications at local level and a complete absence of systemic effects that are typically associated with anti-inflammatory drugs, steroid or surgery [7, 8]. Among HAs used, sodium hyaluronate SUPARTZ® (Seikagaku, Tokyo, Japan) is one of the most used and studied by clinical trials [9–15]. Other products for intra-articular use have been recently introduced for the treatment of OA; at present time there is no definitively effective treatment for this condition, but the very high cost of these therapies, however, fosters to test new treatments that could provide the same benefits at a lower cost. Among these, a medical device MD-Knee, produced by Guna S.p.a., Milan-Italy containing collagen of porcine origin has been investigated. Collagen content in MD-Knee has a molecular weight equal to 300,000 dalton, produced through a process of tangential filtration. It is a pure product, contaminant-free, with standardized chemical and physical characteristics. Collagen is the most abundant protein in the bodies of mammals, accounting for approximately 5–6 % of the body weight of an adult man. About 30 % of total protein mass of higher animals is collagen, found in bones, tendons, joint capsules, muscles, ligaments, teeth, skin and in general in extra-cellular matrix. Porcine tissues have a very high average collagen content, around 50 %. In collagen amino acid content is for Glycine 22.8, Proline 13.8 and Hydroxy-Proline 13 %. The average content of other amino acids is only 3 % (max Glutamic Acid 9.5 %; min Tyrosine 0.4 %). The purpose of an *in-situ* introduction of this device is structural; in fact, mechanical support provided by collagen is an effective natural scaffold support (bio-scaffold). Its degradation in the constituent aminoacids seems to constitute a nutritional support for tissues of the other joint structures [16–19].

The aim of this study is to evaluate the use of collagen MD-Knee versus sodium hyaluronate (SUPARTZ®) in patient with knee OA. The outcome has been clinically assessed through the OMERACT criteria (Outcome Measures in Rheumatology) [20].

## Methods

### Study design and patients

JOINT study is a prospective, double blind, multicentric, randomized clinical trial with active control. The trial was conducted in accordance with the Good Clinical Practice (GCP) and the Declaration of Helsinky; the protocol was approved by the local Ethical Committee (San Pietro Fatebenefratelli Hospital Bioethic Committee). Enrollment started in March 2013 and ended in September 2013. Patients were enrolled and followed in both participating Centers (San Pietro Fatebenefratelli Hospital, Rome, Italy, and Regina Apostolorum Hospital, Albano Laziale, Italy). Only patients affected by symptomatic knee OA were considered eligible for participating in the study. All patients signed an informed consent before entering the study. The randomization list was generated through a high-efficiency system (www.random.org). The list was created by generating eight blocks of eight subjects (1: 1) for a total of 64 enrolled patients. The use of the blocks has allowed to obtain balanced groups during the study. Two groups of subjects were identified; the first group (Group A) consisting of 32 patients has received the investigational product, MD-Knee (Guna S.p.a., Milan, Italy), The second group (Group B), consisting in 32 patients, was treated with SUPARTZ® (Seikagaku, Tokyo, Japan). MD-Knee (injectable ampoules of 2.0 ml) was administered at a dose of two vials for a total of 4 ml via intra-articular injection, once a week for a period of five consecutive weeks; one vial of 2.5 ml sodium hyaluronate (SUPARTZ®) was identically administered.

A total of three visits was performed. During the first one at time T0 (enrollment), the selected patients, after signing the informed consent, were assigned to the experimental group (Group A) or to the reference group (Group B) according to a randomization list. In the same visit the product under investigation was administered. All patients then underwent 1 weekly dosing of MD-Knee or SUPARTZ® for five consecutive weeks; patients were visited 3 months after enrollment (T3 follow-up) and 6 months after the start of the trial (T6 follow-up). The physician performing the intra-articular injection was aware of the product administered, while both physicians evaluating the algo-funcional indices, as well as the patients, were unaware of the product administered.

### Inclusion criteria

In this trial were included male and female subjects who met the following criteria:

- ambulatory adult patients affected by knee OA
- diagnosis according to the ARA (American Rheumatism Association) criteria
- age > 40 years
- disease activity assessed by the Lequesne Knee Index ≥ 7.0 at T0
- disease activity assessed according to the VAS at T0 ≥ 4 cm and persistence of pain in the knee for at least the last 3 months.
- radiological degree II-III according to the Kellgren-Lawrence scale
- patients able to comply with study procedures.

### Exclusion criteria

Patients who met the following criteria were excluded:

- presence of comorbidities (rheumatoid arthritis, spondyloarthritis, connective tissue disease, polymyalgia rheumatica, gout, Paget’s disease, septic arthritis, fractures, osteonecrosis, and fibromyalgia)
- patients with skin or subcutaneous tissue infection in the area of the joint to be treated
- patients who had used oral, parenteral or intra-articular corticosteroids in the 3 months prior to the T0 visit
- patients taking topical analgesics that may interfere with the evaluation of the study
- patients on anticoagulant therapy or suffering from thrombocytopenia and/or coagulopathy
- patients with allergy to products of porcine origins.

### Primary endpoint

At T0 and during the clinical follow-up (FU) at 3 months and 6 months, it was performed the assessment of the physical function according to standardized parameters LKI (Lequesne Knee Index). This clinical trial was set up as a non-inferiority study of MD-Knee compared to sodium hyaluronate (SUPARTZ®) in reducing the LKI score at T3FU in patient with knee OA. At baseline, the average value of the LKI in both groups was assumed to be 7.0 ± 1.1. After 3 months, in Group B (SUPARTZ®), it was expected a reduction of the average value of the LKI to 4.2 ± 1.1 (e.g., a 40 % reduction from baseline). From this value of the LKI score, we accepted as non-inferior a possible value of LKI increased by less than 24 % for the Group A (MD-Knee), and thus the non-inferiority margin (NIM) was set equal to 4.2×1.24 = 5.21, with a standard deviation expected to remain equal to 1.1. Calling D the difference in LKI after 3 months between A and B (equal to 5:21 to 4:20 = 1.01) product, then null hypothesis is H0: D ≥ 1.01, while alternative hypothesis is H1: D <1.01. With these assumptions, the two groups of 29–31 subjects reached a power of 93.8 % in recognizing the non-inferiority using a one-tailed Student *t* test with significance level of α = 0.025. In the case of non-applicability of the Student’s test, it was estimated that the power of the non-parametric analogous test (one-tailed Mann–Whitney *U* test, with a significance level of 0.025) would have been 92.2 %.

### Secondary endpoints

In order to demonstrate the non-inferiority of MD-Knee in reducing pain, the Visual Analogue Scale (VAS) (0–10 cm), the LKI score at T6FU, and the Pain Killer consumption assessment during the course of the study, were also performed.

Questionnaire SF-36 concerning the state of physical and mental health of the subjects, was administered and evaluated for all the patients at T0, T3FU, T6FU. Data were compared with those obtained by the reference group (Group B) and had to comply with the specified threshold for non-inferiority.

Finally, during the investigation period, all events related with intra-articular injection of the investigational product were analyzed.

### Rescue medication

During the study, the only analgesic allowed was Acetaminophen 1000 mg (Pain Killer). Analgesic assumption was reported in a clinical diary.

### Reporting adverse events

All information relating to possible adverse events (AEs), serious adverse events (SAEs) were reported.

### Statistical methods

All variables collected were submitted to the appropriate descriptive analysis, based on their distribution within the sample recruited, assessed by visual inspection of distribution histograms and with the Shapiro-Wilk test for continuous variables, and frequency tables for the categorical variables. The primary endpoint of the possible non-inferiority efficacy in reducing the LKI score (measured at 3 months) in Group A, compared to Group B, was evaluated with Student’s *t* test for independent data, in a one-tailed test, with the significance level of 0.025. The variations of scores between groups obtained from the LKI and the SF36 questionnaire at T0 versus T3FU and versus T6FU were analyzed with repeated measures ANOVA and Bonferroni-adjusted post-hoc test for pairwise comparisons. The changes in the LKI score intra-groups, at T0, versus T3FU and versus T6FU, were analyzed by repeated measures ANOVA plus Bonferroni post-hoc tests.

The change in VAS inter-groups at T0 versus T3FU and versus T6FU, were also analyzed by repeated measures ANOVA, with Bonferroni post-hoc tests. Pain Killer consumption was evaluated with the Mann–Whitney *U* test, after having standardized the values collected. The adverse events in each group were tabulated, and their frequency of occurrence was compared with Fisher’s exact test.

Results of test were considered statistically significant if *p* <0.05, unless for the primary endpoint, for which a one-tailed test with *p* < 0.025 was considered the level of statistical significance. All analyses were carried out with the statistical package Stata/SE 13.1 (The StataCorp LP, 4905 Lakeway Drive, College Station, Texas 77845 USA).

## Results

**Fig. 1:**
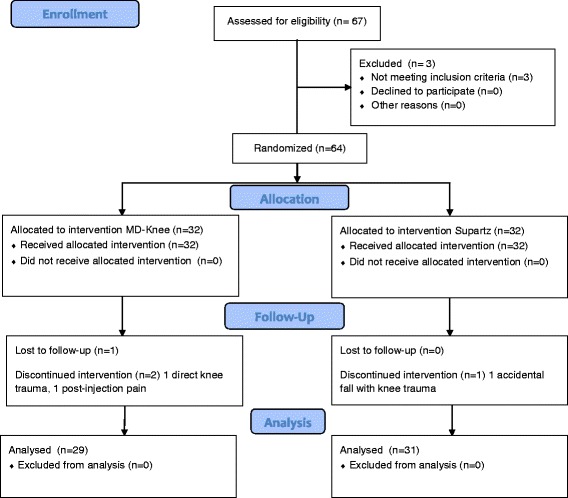
Flow chart reporting patients’ enrollment and study prosecution with drop outs and reasons for drop out

Sixty-seven patients were assessed for eligibility. Three patients were excluded for not meeting the inclusion criteria, as affected by systemic inflammatory arhtritis (2 rheumatoid arthritis, 1 psoriatic arthritis). As reported previously 32 patients in Group A (MD-Knee) and 32 patients in Group B (SUPARTZ®) were enrolled. The patients’ demographic characteristics at T0 are shown in Table 1, evidencing no difference between groups. Women included in Group A were 86.2 and 64.5 % in Group B; mean age was similar in both groups (approximately 69 years). Body Mass Index (BMI) was also similar in both groups, with patients moderately overweight (average BMI approximately 27 kg/m^2^). Kellgren and Lawrence radiological grades II and III were similarly distributed. Function evaluation showed LKI score approximately 12.5. Knee OA symptoms were moderate to severe (average VAS 7.5 cm circa). There was no difference between Group A and Group B in SF36 questionnaire score at T0 (Table 1) and NSAIDs consumption in the previous 3 months (Mann–Whitney *U* test: *p* = 0.8439) (data not shown). Three patients in the MD-Knee arm and one patient in the SUPARTZ arm dropped out before study conclusion. In the MD-Knee one patient experienced joint pain after the second intra-articular injection of MD-knee and decided to withdraw from the study, with knee pain regressing in 1 day without the need of any medicaments and no signs of joint effusion/inflammation, one patient experienced a direct blunt trauma in the knee after the second MD-knee injection and was then excluded from study prosecution and one patient was lost to followup. In the SUPARTZ group, one patient experienced an accidental fall with multiple contusions, involving studied knee, after the third injection of SUPARTZ and was then excluded from the study (Fig. 1).

**Table 1:** Patients’ demographic characteristics at T0

	Group A (MD-Knee) = 32	Group B (SUPARTZ®) = 32
Age (years ± SD)	69.41 ± 8.42	69.97 ± 9.5
Women, n (%)	25 (86.2 %)	20 (64.5 %)
BMI (kg/m^2^)	27.20 ± 3.78	27.3 ± 3.56
Kellgren and Lawrence, n (%)Grade II (%)	15 (51 %)	17 (55 %)
Kellgren and Lawrence, n (%)Grade III (%)	14 (49 %)	14 (45 %)
LKI ± SD	12.48 ± 2.63	12.6 ± 3.48
SF36 ± SD	91.41 ± 20.01	93.07 ± 17.3
Pain VAS (cm) ± SD	7.67 ± 1.41	7.42 ± 1.35

Data are mean ± *SD* Standard Deviation unless otherwise indicated. *BMI* Body Mass Index, *LKI* Lequesne Knee Index, *VAS* Visual Analogue Scale

### Primary endpoint

#### Non-inferiority of LKI score at T3FU

**Fig. 2:**
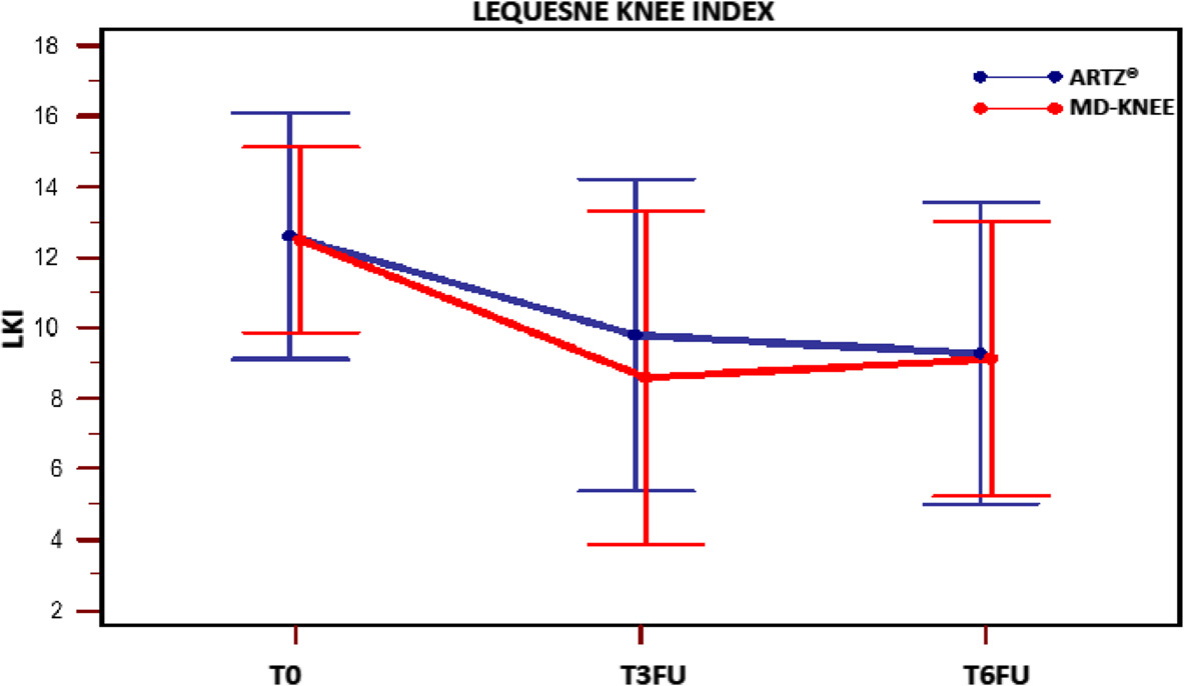
Variation of LKI (Lequesne Knee Index) score intra-subject and inter-groups at time T0; T3FU; T6FU

LKI score was the same in the two groups at T0 (Student’s *t* test: *p* = 0.8871) and at T3FU (Student’s *t* test: *p* = 0.3302). Observed difference intra-groups (T3FU mean LKI - T0 mean LKI) was larger in absolute value in Group A (MD-Knee) than in Group B (SUPARTZ®), but this difference was not significant (Welch’s test: *p* = 0.3683). The mean difference between Group A and Group B was equal to − 3.7778 - (−2.9483) = −0.8295, and the confidence interval (CI) 95 % of this difference ranged from − 2.6737 to +1.0147, while the standard error was 0.9110. Since the variances at T3FU did not differ between groups (F-test: *p* = 0.7540), the variance and the standard error pooled were used, keeping the non-inferiority limit by 24 % compared to the value measured in Group B, as established by the protocol. The precise difference between the average score was then − 1.2005, while the interval difference, taking into account the degrees of freedom of the system (dof = 54), was included with the 99 % confidence between − 4.47004 and +2.06904, and since the non-inferiority limit calculated for the differences between averages was +2.3503, we could conclude that the treatment with MD-Knee was not inferior of the treatment with SUPARTZ®, with a confidence level higher than 99 %. Table 2 – Fig. 2

**Table 2:** Data are mean ± SD (Standard Deviation) at T3FU and T6FU with *p* value

	T3 FU			T6 FU		
	Group A (MD-Knee)	Group B (SUPARTZ®)	*P*-value	Group A (MD-Knee)	Group B (SUPARTZ®)	*P*-value
LKI ± SD	8.59 ± 4.71	9.79 ± 4.43	0.33	9.12 ± 3.89	9.28 ± 4.28	0.621
Pain VAS ± SD	5.26 ± 2.52	5.13 ± 2.41	NE	5.42 ± 2.69	4.43 ± 2.63	0.275
SF36 ± SD	99.15 ± 8.95	101.32 ± 6.37	NE	88.37 ± 28.83	92.07 ± 23.37	0.462

*NE* Not evaluated

### Secondary endpoints

**Fig. 3:**
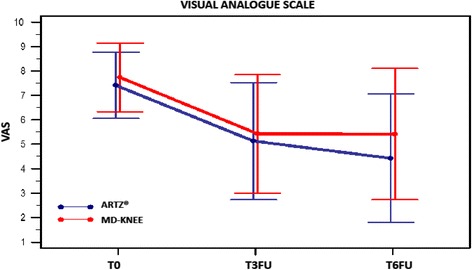
Visual Analogue Scale (VAS) at time T0; T3FU; T6FU for Group ARTZ® and in Group MD-Knee
- *SF36 questionnaire*
  Repeated measures ANOVA of SF36 questionnaire total score showed a significant change intra-patient (F-test: *p* = 0.005, without assuming the sphericity of the data), while no changes were observed due to treatment (F-test: *p* = 0.462) at T3FU and T6FU. Table 2 - Fig. 4

- *LKI variation inter- groups at T0* versus *T6FU*
  Repeated measures ANOVA of LKI showed a significant reduction intra-patient (F-test: *p* <0.01), even assuming the non-sphericity of the data. It was observed no change due to membership in the treatment group (F-test: *p* = 0.621). Table 2 - Fig. 2
- *Pain VAS*
  Repeated measures ANOVA of VAS showed a intra-patient highly significant variation, while inter-groups variation (*p* = 0.275) and interaction (group x factor) (*p* = 0.447) are not significant. Therefore VAS variation does not seem to depend on the administered treatment. Table 2 – Fig. 3

**Fig. 4:**
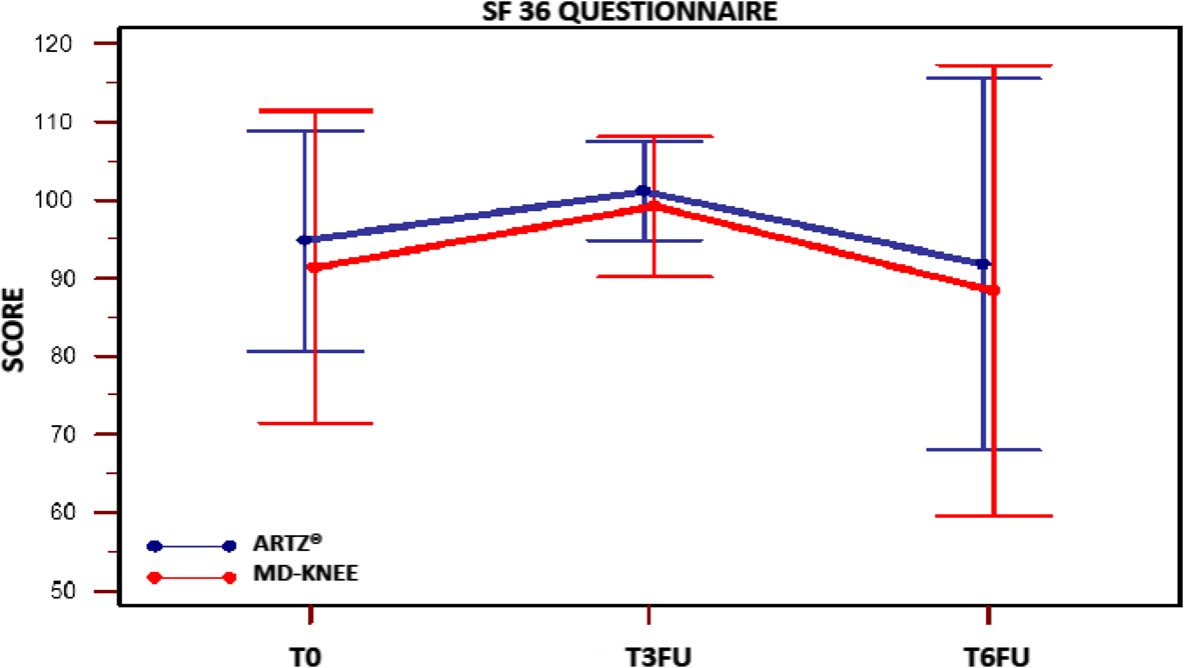
SF36 questionnaire. Score at time T0; T3FU; T6FU for Group ARTZ®and Group MD-Knee
- *Pain Killer consumption (Rescue Medication)*
  Including dropouts, Acetaminophen was used by 13 of 29 patients (44,8 %) in Group A (MD-Knee) and by 12 of 31 patients (38,7 %) in Group B (SUPARTZ®). Acetominophen consumption during the trial did not change in the two Groups, considering both “only users” and “all patients” (Mann–Whitney test U: *p* = 0.2198 e *p* = 0.9348, respectively). Fig. 5a, b.

**Fig. 5:**
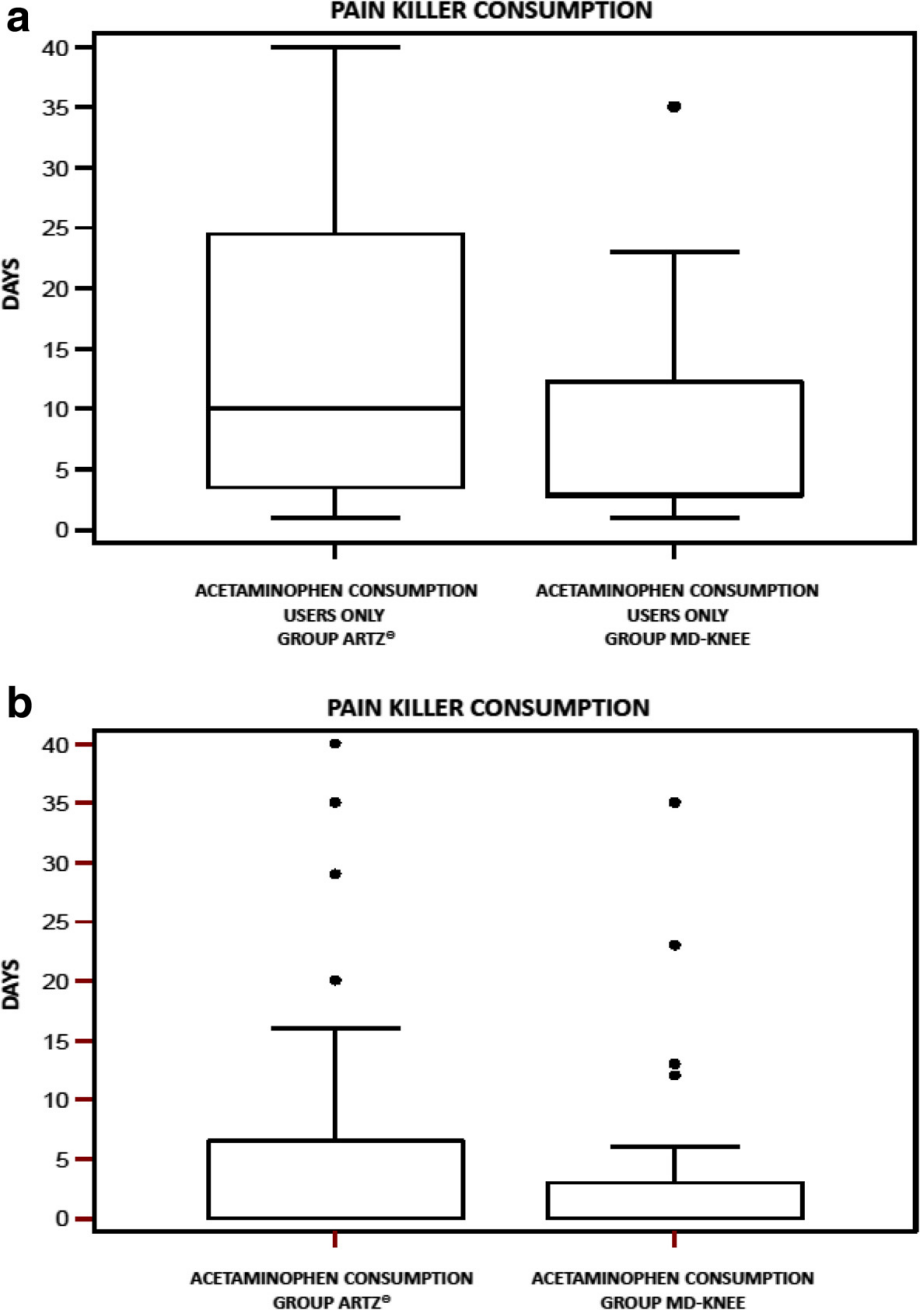
**a** Pain Killer consumption (days), in users only, in both groups. **b** Pain Killer consumption (days),in all subject, in both groups

#### Safety

Adverse events (AE) observed by the investigators or reported by the patients spontaneously or following a non-leading question, were investigated. Treatment with MD-Knee and with SUPARTZ® for up to 6 months was generally well tolerated. No systemic adverse events and septic complication were observed. Only one subject discontinued for a moderate post-injection reaction in Group A (MD-Knee) but symptoms disappeared without the need of medication. No joint effusion events were observed throughout the entire followup of patients in both groups.

## Discussion

Intra-articular (IA) therapy in the treatment of OA knee is widespread in clinical practice, although much debated by the evidences of the most recent international recommendations. The IA therapy may consist of corticosteroids, of high or low molecular weight (MW) HA, of polynucleotited, pletelet-rich-plasma (PRP) or other substances including collagen extracts. Recent scientific evidences suggest that injecting treatment with porcin collagen could provide interesting improving clinical performances [16–19]. However IA therapy should be considered with the complex management of OA, such as medical and non- medical interventions. I The collagen administered at intra-articular level could stimulate and promote the healing process of the cartilage matrix, which is injured in the course of osteoarthritis, as demonstrated in animal models [17]. Collagen can promote repair processes of the cartilage matrix, interrupting the degenerative process and articular damage, which causes inflammation and pain.

In this double-blind, randomised, active-controlled clinical trial in patients affected by knee OA, five intra-articular injections of MD-Knee or sodium hyaluronate administered weekly are equally able to improve function and reduce pain after 3 months till at least 6 months by the end of treatment. As shown VAS and LKI improved at T3FU and T6FU. SF36 questionnaire, and pain killer consumption did not change in both groups confirming non-inferiority hypothesis. We have to acknowledge limitations of this study. In this non inferiority prospective randomized controlled double blind study, for bio-ethical reasons, we have no placebo arm, therefore the confrontation was made between two active arms only. Also, followup time was limited to 6 months only, while longer followups are recommended for chronic pathologies such as OA.

Future trials should investigate the proportion of OARSI/OMERACT criteria responders which might be useful for an indirect comparison with other local or systemic treatments; also the effect on the progression of tissue damage could be looked into. The lack of a placebo control group is to be expected when IA injections of hyaluronic acid products are routinely used in clinical practice. A further issue concerns the nature of placebo for IA injections, ie. use of saline solution or sham injection. For these ethical and methodological reasons, it has been considered correct to compare MD-Knee with a marketed product, such as SUPARTZ®, that has been widely used in clinical practice and proved effective in previous studies. Moreover the lack of comparison with patients treated with other kind of HA (as high MW or cross-linked HA) doesn’t allow us to draw final conclusion about the range of efficacy of MD-Knee Guna Medical Device, since the clinical equivalence of all HA products is not clear. Nevertheless a favourable feature of this trial is assessing OA knee symptoms effectiveness through several different measures, providing a wide clinical evaluation. Beside all Lequesne’s items showing a similar advantage for both groups. Further studies are warranted in order to verify whether the symptomatic effect of MD-Knee is associated with a halting of knee OA progression.

## Conclusion

This trial shows that MD-Knee preparation is effective on knee OA symptoms over 6 months after a 5-week injection course, and it is equally effective in improving clinical performance as assessed through LKI, VAS, SF36 questionnaire and Pain Killer consumption, as the reference HA formulation.

MD-Knee and SUPARTZ® were equally well tolerated both locally and at a systemic level, therefore showing a satisfactory safety profile.

The reduced cost of MD-Knee compared to low or high MW HA could allow wider use of IA therapy, resulting in a NSAIDs intake reduction, as well as social cost reduction due to working days lost and caregivers time off work.
